# Identification of *Nigrospora oryzae* Causing Leaf Spot Disease in Tomato and Screening of Its Potential Antagonistic Bacteria

**DOI:** 10.3390/microorganisms13051128

**Published:** 2025-05-14

**Authors:** Jun Zhang, Fei Yang, Aihong Zhang, Qinggang Guo, Xiangrui Sun, Shangqing Zhang, Dianping Di

**Affiliations:** 1 Key Laboratory of Integrated Pest Management on Crops in Northern Region of North China, Plant Protection Institute, Hebei Academy of Agriculture and Forestry Sciences, Ministry of Agriculture and Rural Affairs, China/IPM Innovation Center of Hebei Province, International Science and Technology Joint Research Center on IPM of Hebei Province, No. 437 Dongguan Street, Baoding 071000, China; zhangjunml@haafs.org (J.Z.); feiyang_987@163.com (F.Y.); zhangaihong08@163.com (A.Z.); gqg77@163.com (Q.G.); sxr1985@163.com (X.S.); 2 Tangshan Academy of Agricultural Sciences, Tangshan 063000, China; zhangshangqing85@163.com

**Keywords:** *Nigrospora oryzae*, phytopathogenic fungi, tomato, *Bacillus velezensis*, transformation system

## Abstract

Tomato is a widely cultivated vegetable crop worldwide. It is susceptible to various phytopathogens, including fungi, bacteria, viruses, and nematodes. In 2024, an unknown leaf spot disease outbreak, characterized by distinct brown necrotic lesions on leaves, was observed in tomato plants in Yunnan Province, China. Through rigorous pathogen isolation and the fulfillment of Koch’s postulates, it was proved that the fungal isolate could infect tomato leaves and cause typical symptoms. The pathogen isolated from tomato leaves was identified as *Nigrospora oryzae* based on its morphology and using a multilocus sequence analysis method with the internal transcribed spacer gene (ITS1), beta-tubulin gene (TUB2), and translation elongation factor 1-alpha gene (TEF1-α). This represents the first documented case of *N. oryzae* infecting tomatoes in the world. Given the damage caused by *N. oryzae* to tomato plants, we explored biocontrol methods. Through a dual-culture assay on PDA plates, *Bacillus velezensis* B31 demonstrated significant biocontrol potential, exhibiting strong antagonistic activity toward *N. oryzae*. In addition, we developed a polyethylene glycol (PEG)-mediated transformation system that successfully introduced pYF11-GFP into the protoplasts of *N. oryzae*. This achievement provides a foundation for future genetic manipulation studies of *N. oryzae*.

## 1. Introduction

The tomato (*Solanum lycopersicum*) is considered one of the most economically important vegetable crops, widely cultivated in multiple countries and regions due to its nutritional value. It is a cornerstone of agricultural systems, contributing to food security and livelihoods worldwide. In China, with a large population and limited cultivated land, tomato cultivation is of great significance for ensuring the agricultural product supply and maintaining the existing basic farmland area [1]. However, tomato production faces persistent challenges from biotic stressors, particularly diseases, which can severely impact the yield and quality [2].

Tomato diseases are caused by diverse pathogens, each with distinct biological and epidemiological traits. For instance, late blight (*Phytophthora infestans*) can result in a 100% yield loss under hyperthermic and high-humidity conditions [3]. In addition, notorious tomato pathogens also include the necrotrophic fungi *Botrytis cinerea*, *Alternaria solani*, *Cladosporium fulvum,* and *Fusarium oxysporum f. sp. lycopersici* [4]. Foliar and vascular diseases, such as leaf spot and wilt syndromes, caused by these fungi represent a widespread and persistent challenge in tomato cultivation due to their agricultural significance and high incidence globally [5].

In 2024, a novel foliar disease emerged in greenhouse-cultivated tomatoes in Yunnan Province, China, characterized by distinct interveinal chlorosis progressing to necrotic lesions. Field surveys indicate a disease incidence of 20–30%. Initial symptoms manifest as irregular chlorotic patches between leaf veins, followed by central browning and necrosis, ultimately forming elliptical lesions (3–8 mm in diameter) with defined margins. Consequently, the infection induces premature leaf shedding and an impaired photosynthetic capacity, leading to a significant decrease in yield. Preliminary morphological analyses suggest that the causal agent is *Nigrospora oryzae*, a species within the genus *Nigrospora*, which belongs to the ascomycete fungi and has a wide distribution and host range [6].

To date, *Nigrospora* species are widely documented as plant pathogens of many economically important crops, fruits, and ornamentals. For example, leaf spot as well as black rot were observed in kiwifruit [7], leaf spot in olives [8], leaf blight in elephant grass [9], and reddish-brown spot disease in red-fleshed dragon fruit [10]. It is worth noting that *N. oryzae* also causes panicle branch rot disease on *Oryza sativa* [11]. Similarly, Wang et al. observed the morphology of the isolated rice leaf streak pathogen and identified it as *N. oryzae* by combining the ITS1, TEF1-α, and TUB2 gene sequences. To sum up, these diseases have been causing serious economic losses in agricultural production and forestry. However, it remains unclear whether *N. oryzae* can infect tomatoes, and the pathogenesis of *N. oryzae* and host–pathogen interactions remains largely elusive.

Several studies have reported on the control of *N. oryzae*. Current control strategies for *N. oryzae* heavily rely on chemical interventions. However, the frequent use of chemical pesticides is associated with considerable environmental, health, and safety concerns [12]. In contrast, biological control methods, particularly the use of antagonistic bacteria, have shown promise. For instance, *Bacillus subtilis* NCD-2, HMB19198, and *Bacillus velezensis* B31 have demonstrated strong antagonistic effects against phytopathogenic fungi exhibiting broad-spectrum antifungal activities [13,14,15]. Despite these findings, the application of biocontrol bacteria as a biocontrol method to antagonize *N. oryzae* is limited.

In order to identify the pathogen that caused the tomato black–brown necrotic spots disease in Yunnan Province and obtain potential antagonistic bacteria for the prevention and control of the disease, we conducted the following work in this study: (1) to isolate and identify the causal pathogen; (2) to confirm its pathogenicity; (3) to establish a genetic transformation method for *N. oryzae-YN*; and (4) to evaluate the antagonistic effect of NCD-2 on *N. oryzae*. This work is of great significance for the research and biological control of leaf diseases in tomatoes.

## 2. Materials and Methods

### 2.1. Pathogen Isolation and Culture Conditions

Symptomatic tomato leaves were collected from Yunnan Province, China. The collected samples were taken to the laboratory and immediately processed. For pathogen isolation, a small section (about 2 mm × 2 mm) taken at the border between the infected and healthy parts of the diseased leaf was aseptically cut and surface-sterilized in 75% ethanol for 30 s, washed twice with sterile water, then immersed in 3% sodium hypochlorite solution for 1 min, and rinsed three times in sterile water. Leaf sections were inoculated onto potato dextrose agar (PDA) plates containing 50 µg/mL ampicillin and incubated at 25 °C in the dark for 5 days. Pure mycelia were obtained by hyphae tip separation. Mycelium from the periphery of the colony was picked with a sterilized toothpick and subcultured onto new PDA plates. This subculture process was repeated until pure colonies were obtained.

### 2.2. Strains Used in This Study

*Magnaporthe oryzae* Guy11 was preserved in our laboratory. For vegetative growth, 2 mm × 2 mm mycelial blocks were placed onto freshly prepared PDA. *Bacillus subtilis* NCD-2, HMB19198, and *Bacillus velezensis* B31 were isolated and preserved by the Institute of Plant Protection, Hebei Academy of Agricultural and Forestry Sciences, China. Strains NCD-2, B31, and HMB19198 were stored at −80 °C in Luria–Bertani broth (LB) with 30% glycerol (vol/vol). Fresh bacterial cultures were retrieved from frozen stocks before each experiment and streaked onto LB agar plates. After overnight incubation at 37 °C, single colonies were picked and inoculated into 50 mL of LB broth. The cultures were grown at 30 °C with shaking at 150 rpm for 24 h. Then, the cell density of the cultures was measured using a spectrophotometer at 600 nm (OD600), and the cultures were adjusted to a concentration of 1 × 10^6^ CFU/mL with sterile phosphate-buffered saline (PBS) to prepare the bacterial suspension for the in vitro antagonism assay.

### 2.3. Assays for Pathogenicity and Morphological Observations

Conidia were collected from *N. oryzae* isolated from Yunnan grown on V8 medium for 7 days and re-suspended in 0.2% (*w*/*v*) gelatin solution to a concentration of 5 × 10^4^ spores/mL. For spraying assay, five-week-old tomato seedlings (*Solanum lycopersicum* Jingfan309) were sprayed with 4 mL of the conidial suspension of *N. oryzae-YN* and kept in a growth chamber at 25 °C with 90% humidity in the dark for the first 24 h, followed by a 12 h light and 12 h dark cycle. Lesion formation was checked daily and photographed after seven days of inoculation.

### 2.4. Genomic DNA Extraction and PCR Amplification

The genomic DNA was obtained by using the Plant Genomic DNA kit (Tiangen, Beijing, China). The primer pairs ITS1/ITS4 were used to amplify the internal transcribed spacer region (ITS1), Bt2a/Bt2b were used to amplify the beta-tubulin gene (TUB2), and TEF1-728F/EF2 were used to amplify the translation elongation factor 1-alpha gene (TEF1-α). The primers are summarized in Table 1. The obtained sequences by PCR were sent to Sangon Biotech (Beijing, China) for sequencing.

### 2.5. Phylogenetic Analysis

Several reference sequences of the *N. oryzae* genus from GenBank (http://www.ncbi.nlm.nih.gov, accessed on 25 March 2025) used in the phylogenetic analysis and GenBank accession numbers obtained in this study are listed in Table 2. The phylogenetic tree was constructed using the Maximum Likelihood method based on the Tamura-Nei model in MEGA-11 software, with node support assessed through 1000 bootstrap replicates.

### 2.6. Polyethylene Glycol (PEG)-Mediated Transformation of N. oryzae

According to Liu’s method with small modification, we established a genetic transformation method for *N. oryzae* [16]. *N. oryzae* mycelia were inoculated into liquid CM medium and incubated with shaking at 28 °C for 36 h. Subsequently, mycelia were harvested by filtration through Miracloth (475855-1R, Merck Millipore (Darmstadt, Germany), made in USA, sourced from Casmart in China) and resuspended in 30 mL of enzyme buffer containing 0.7 M NaCl and 0.1% (*w*/*v*) lysing enzyme (L3768, Sigma-Aldrich, St. Louis, MO, USA). The suspension was incubated at 30 °C for 4 h with gentle shaking (70 rpm) to digest cell walls and release protoplasts. The mixture was filtered through Miracloth to remove undigested mycelia and debris. The filtrate was centrifuged at 3600 rpm for 10 min to collect the protoplasts. Purified protoplasts were washed twice with STC buffer (1.2 M sorbitol, 10 mM Tris-HCl, 50 mM CaCl_2_, pH 7.5) and concentrated to 1 mL in 1 × STC buffer. For transformation, 100 μL protoplast suspension was combined with 10 μg plasmid DNA in sterile tubes, gently mixed by tapping, and incubated at 25 °C for 25 min. Following addition of 1 mL PTC solution (60% PEG4000 in 1 × STC), the mixture was incubated for another 25 min at 25 °C. Then, 8 mL of STC buffer was added to the tube and then incubated at 30 °C for 2 h with gentle shaking (70 rpm). Finally, the protoplast suspension was mixed in a 50 mL conical tube with 40 mL of TB3 medium agar containing an appropriate antibiotic. The mixture was then quickly poured into a clean Petri dish. All plates were incubated statically at 28 °C for 7 days until colony emergence.

### 2.7. The Extracellular Laccase and Peroxidase Activity of N. oryzae

CR (Congo red, C804149, Macklin, made in Shanghai, China, sourced from Casmart in China) can be used to detect secreted peroxidases as it can be hydrolyzed by fungal peroxidases [17]. Similarly, 2,2′-azino-bis (3-ethylbenzothiazoline-6-sulfonic acid) (ABTS, A800764, Macklin, made in China, sourced from Casmart in China) is utilized to detect extracellular laccase activity; it turns green–blue when oxidized by laccase [18]. In order to determine whether *N. oryzae-YN* has extracellular laccase and peroxidase activity, we inoculated *N. oryzae-YN* and Guy11 (*Magnaporthe oryzae*) strains onto PDA plates containing 0.2 mM ABTS and 200 μg/mL CR. Then, we observed whether a golden halo (indicating CR degradation) or an oxidized dark purple halo (indicating ABTS degradation) was generated around the mycelium plug after incubation at 28 °C for 2 days.

### 2.8. Epifluorescence Microscopy Observation

pYF11-GFP (bleomycin resistance), which is preserved in our laboratory, was transformed into *N. oryzae* by PEG-mediated transformation method. Vegetative hyphae, conidia-expressing *GFP* genes, were incubated under appropriate conditions. Epifluorescence microscopy was performed using a Leica TCSSP8 (63× oil) microscope.

### 2.9. In Vitro Antagonism Against N. oryzae

The antagonism ability of each antagonistic strain screened from the previous study was determined by dual-culture assay on PDA plates [19]. In general, we took a 6 mm agar–mycelium plug obtained from *N. oryzae-YN* cultured on PDA medium for 5 days and placed it in the center of a new PDA plate. The plate was then incubated at 27 °C in the dark for 1 day. Next, 0.5 µL of the antagonistic bacterial suspension (1 × 10^6^ CFU/mL) was added dropwise to 4 areas of the PDA plate, each positioned 1 cm from the edge of the plate. As a control, the same volume of sterile distilled water was added dropwise to the corresponding positions on another PDA plate. After that, all plates were cultured at 27 °C in the dark for 6 days. The colony radius of *N. oryzae-YN* was measured in mm using a vernier caliper, with the results retained to two decimal places. The experiment was repeated twice, with three replicates for each treatment.

### 2.10. Statistical Analysis

Mean and standard deviation (SD) were calculated from at least three independent replicates. The significance of differences between samples was statistically evaluated using SD and analysis of variance (ANOVA) in GraphPad Prism 8.0.1, as described previously [20].

### 2.11. Accession Numbers

Genes used in this study are able to be found in the GenBank database (https://www.ncbi.nlm.nih.gov/protein/, submitted on 5 March 2025, accessed on 25 March 2025) using the accession numbers in Table 3.

## 3. Results

### 3.1. Plant Disease Symptoms and Pathogenicity Test

**Figure 1:**
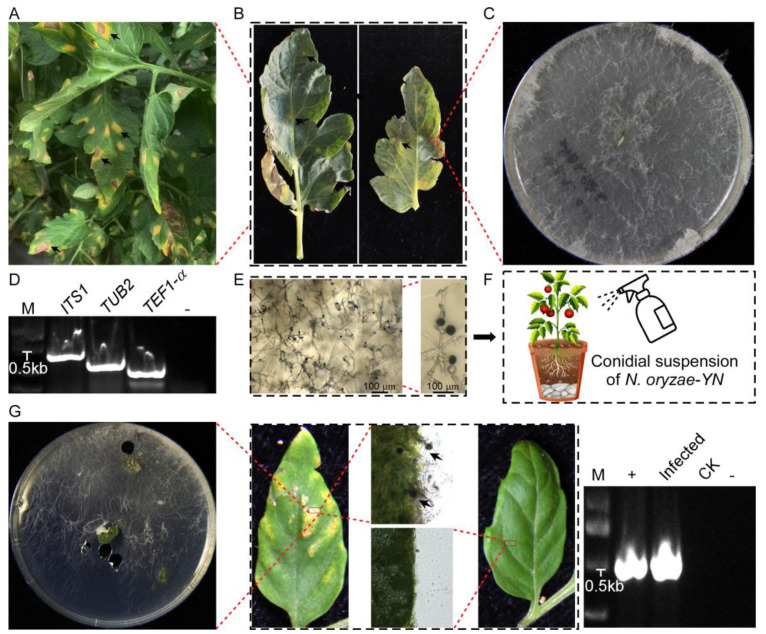
Symptoms of disease in tomato and pathogenicity test. (**A**) Field symptoms. (**B**) Diseased branches near the top of the tomato. (**C**) Colony on PDA medium after 7 days. (**D**) The ITS1, TUB2, and TEF1-α gene of *N. oryzae-YN* obtained sequences by PCR. M: DNA Marker 2000. (**E**) Conidia and hyphae observed with optical microscope. (**F**) Spraying assay: conidial suspensions of *N. oryzae-YN* were sprayed onto leaves of five-week-old tomato seedlings (*Solanum lycopersicum* Jingfan309), The arrow represents the conidia generated in Figure 1E, which are further utilized in the Spraying assay. (**G**) Observed the disease symptoms on tomato leaves after inoculating them with conidial suspensions of *N. oryzae-YN*, and photographed at 7 days after inocubation (dai) (top panels). Fungal growth assay: the diseased tomato leaves were surface-sterilized and incubated in a chamber for 24 h, and examined under a microscope. For pathogen isolation, leaf sections were inoculated onto PDA medium. To further confirm the identity of *N. oryzae-YN*, the *ITS* gene obtained sequences by PCR. In order to confirm the identity of *N. oryzae-YN* again, the sequence of *ITS* gene was obtained through PCR (bottom panels).

The disease was discovered in greenhouse-cultivated tomatoes in Yunnan Province, manifesting distinct foliar symptoms. The initial infection presented as irregular chlorotic lesions (Figure 1A), which progressively expanded into coalescing necrotic patches with characteristic black discoloration (Figure 1B). Advanced disease progression led to extensive leaf chlorosis and premature abscission, significantly reducing plant growth and productivity.

Through systematic pathogen isolation from surface-sterilized lesion margins, a fungal strain was consistently isolated as the sole pathogen. When cultured on PDA medium, the isolate initially formed white mycelial colonies that gradually developed dark gray–black pigmentation in the central regions as they matured (Figure 1C). A microscopic examination showed hyaline to pale brown hyphae with smooth surfaces, regular branching, and distinct septa. Conidiogenesis generated numerous solitary conidia with dark brown to black pigmentation, smooth walls, an aseptate structure, and a consistent diameter of 11–15 µm (Figure 1E).

**Figure 2:**
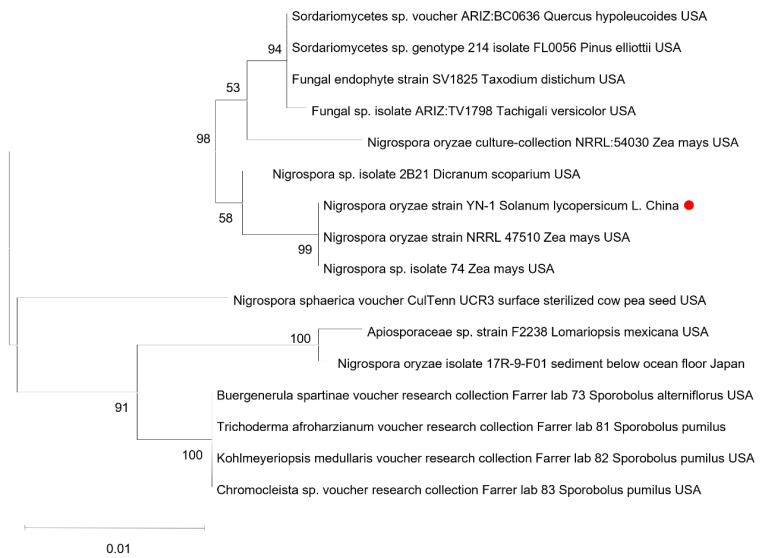
Phylogenetic tree obtained from maximum likelihood method based on the ITS sequences of *N. oryzae*. The phylogenetic tree was constructed using the Maximum Likelihood method based on the Tamura-Nei model in MEGA-11 software, with node support assessed through 1000 bootstrap replicates. The red dot indicates the *N. oryzae* isolate from Yunnan.

To confirm the identity of the pathogen through the molecular characterization, amplification, and sequencing of the ITS1, TUB2, and TEF1-α gene with specific primer pairs (Table 1), yielded sequences (Figure 1D) were deposited in GenBank (GenBank accession nos. PV382401, PV230471, and PV274065, respectively). A BLAST 2.15.0+ (https://blast.ncbi.nlm.nih.gov/Blast.cgi, accessed on 7 March 2025) analysis of the sequenced loci indicated that the ITS1, TUB2, and TEF1-α sequences had, respectively, 99.43%, 89.60%, and 72.46% identity with the *N. oryzae* sequences (GenBank accession nos. EU272488.1, OP450803.1, and KY019386.1) (Table 3). The result, confirmed by single-gene phylogenetic analysis (Figure 2), reliably proved that the isolates were *N. oryzae*.

The pathogenicity of the *N. oryzae-YN* strain to tomato plants was validated through standardized foliar inoculation procedures. Five-week-old tomato seedlings (*Solanum lycopersicum* cv. Jingfan309) were inoculated with a calibrated conidial suspension (5 × 10^4^ spores/mL) prepared from fungal cultures under aseptic conditions (Figure 1F). Within 14 days post-inoculation, characteristic disease symptoms developed exclusively on treated leaves, manifesting as circular-to-irregular necrotic lesions (3–5 mm in diameter) with distinct chlorotic halos, while control plants remained completely asymptomatic. Repeated independent biological experiments demonstrated consistent symptom progression and disease severity, and the fungal pathogen was successfully reisolated from symptomatic tissues and confirmed as *N. oryzae-YN* through ITS1 sequencing analysis (Figure 1G). This pathogenicity assessment fully satisfied Koch’s postulates, thereby establishing, for the first time, that *N. oryzae* can function as a causal agent of tomato disease. 

### 3.2. Morphological Characteristics of Conidia

**Figure 3:**
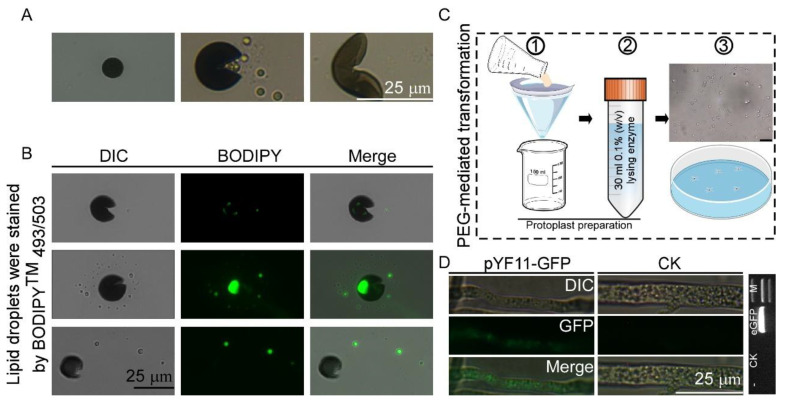
Morphological characteristics of conidia and establishment of genetic transformation methods for *N. oryzae*. (**A**) Conidial suspensions of *N. oryzae* were drop-inoculated onto a cover glass to induce appressorium formation. (**B**) Lipid droplets were stained by BODIPYTM 493/503 at different time points and examined under a confocal microscope. Bar = 10 μm. (**C**,**D**) Established PEG-mediated protoplast transformation system of *N. oryzae* and it was confirmed that the green fluorescent signal was detected in the *N. oryzae* transformants by PCR as well as under the microscopic analysis. Bar = 10 μm. M: DNA Marker 2000. *eGFP* = 717 bp. ① Filter and collect the mycelium. ② Conduct enzymatic hydrolysis. ③ PEG-mediated protoplast transformation.

Phytopathogenic and mycorrhizal fungi often penetrate living hosts by using appressoria and related structures [21], and physical cues of an inductive surface, such as the hardness and hydrophobicity, are required for appressorium formation [22]. In order to observe the appressorium formation of *N. oryzae-YN*, a conidial suspension (5 × 10^4^ spores/mL) of *N. oryzae-YN* was drop-inoculated onto plastic cover slips (hydrophobic) and incubated under humid conditions at 25 °C. We found that no appressoria formation was observed by *N. oryzae-YN* on plastic cover slips, but instead, a number of conidia cracked irregularly, releasing granular liposomes after 24 h of incubation. To further determine the substances released during the cracking process, we stained the released granular substances with BODIPY^TM^ 493/503 at different time points and examined them under a confocal microscope. The results showed that these substances could be stained with green fluorescence (Figure 3A,B).

### 3.3. Establishment of Genetic Transformation Methods for N. oryzae

An optimized PEG-mediated protoplast transformation system was developed for *N. oryzae* through the systematic refinement of protoplast preparation protocols and genetic transformation methodologies. The molecular characteristics of the putative transformant revealed the successful integration of the enhanced green fluorescent protein (*eGFP*) genes, which was confirmed by the PCR amplification of target sequences and subsequent DNA sequencing validation. Fluorescence microscopy analysis showed the functional expression of heterologous proteins, and distinct green fluorescent signals were detected in the hyphal compartments of the transformed strain (Figure 3C,D). This transformation scheme has been rigorously validated through multiple experiments, confirming its reliability in the genetic manipulation of *N. oryzae*. It will accelerate the studies on the gene functions of *N. oryzae*, especially those genes associated with the pathogenesis of this fungus in tomatoes.

### 3.4. N. oryzae-YN Lost the Extracellular Laccase and Peroxidase Activity

**Figure 4:**
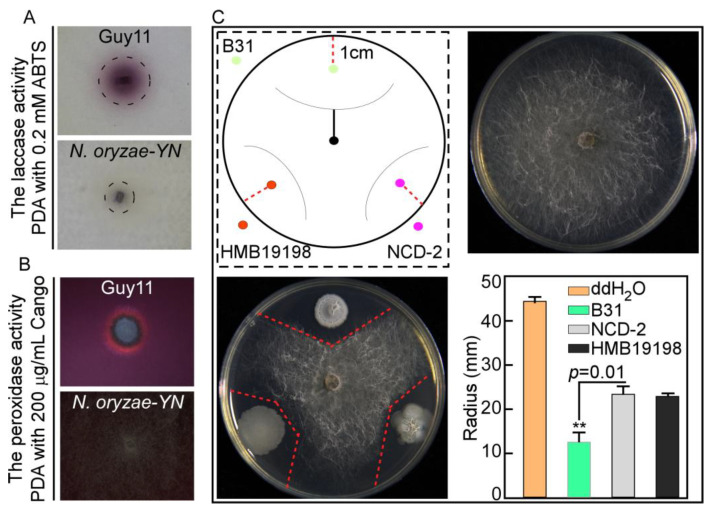
Detection of extracellular laccase and peroxidase activity of *N. oryzae-YN* and screening of its biocontrol bacteria. (**A**,**B**) *N. oryzae-YN* and Guy11 strains inoculated onto PDA contain 0.2 mM ABTS and 200 μg/mL CR and observe the purple degradation zones and golden halo formed around colonies after 2 days or 7 days incubation at 28 °C. (**C**) Schematic diagram of dual culture assay, central position represents *N. oryzae-YN*, the three directions represent bacterial strains, and the red line represents the colony radius of *N. oryzae-YN*. As a control, the same volume of sterile distilled water (ddH_2_O) was added dropwise to the corresponding positions on another PDA plate, B31, NCD-2, and HMB 19198, and they represent *Bacillus velezensis* B31, *Bacillus subtilis* NCD-2, and *Bacillus subtilis* HMB19198, respectively. Error bars are standard deviations from three biological repeats and asterisk indicates significant differences at *p* < 0.05.

Pathogens have evolved ingenious mechanisms to overcome host immunity by eliminating ROS or suppressing host ROS generation by secreting a large number of catalases, peroxidases, extracellular laccase, and effectors [20]. The extracellular laccase and peroxidase activity of *N. oryzae-YN* was evaluated through chromogenic reactions with ABTS and Congo Red (CR) substrates, respectively. When cultured on PDA medium supplemented with 0.2 mM ABTS and 200 µg/mL CR, the *N. oryzae-YN* strain exhibited significantly reduced enzymatic activities compared with the Guy11 control strain. Specifically, no dark purple oxidative staining was observed around *N. oryzae-YN* colonies in the laccase assay, and the golden halo formation was smaller than that of Guy11 in the peroxidase assay (Figure 4A,B). This phenotypic difference indicates significantly reduced extracellular laccase activity and substantially impaired peroxidase functionality in the *N. oryzae-YN* strain.

### 3.5. Screening of Antagonistic Bacteria

Three strains of *Bacillus subtilis* NCD-2, HMB19198, and *Bacillus velezensis* B31, which are known for their strong antagonism toward phytopathogenic fungi, were selected to evaluate their antimicrobial effects on *N. oryzae*. In the dual culture assay on PDA plates, the plates treated with *Bacillus velezensis* B31 had the smallest fungal colony radius of only 13.32 mm, which shows an extremely significant difference compared to the control (44.25 mm) (Figure 4C). These results indicated that B31 exhibited the strongest antagonistic activity against *N. oryzae* among the three biocontrol bacteria.

## 4. Discussion

The tomato is the world’s second-most widely cultivated vegetable. However, it faces persistent threats from diseases across all production systems. Even crops grown in protected environments such as greenhouses and high tunnels remain vulnerable to infection. Similarly, those in small-scale home gardens are also at risk. Among the most significant fungal diseases impacting tomato production are late blight, *Sclerotinia rot*, *Fusarium wilt*, *Fusarium crown*, and root rot [23]. These pathogens continue to cause substantial economic losses in both open-field and protected cultivation systems, with their persistent presence remaining a primary constraint on global tomato productivity [24]. In Yunnan Province, China, an emerging foliar disease affecting tomato plants (*Solanum lycopersicum*) has been identified as causing substantial agricultural losses through severe leaf damage. Field surveys documented characteristic symptoms including chlorosis and necrotic lesions with associated economic impacts on local tomato production. Through integrated morphological characterization and molecular phylogenetic analysis, we conclusively identified *Nigrospora oryzae* as the causal agent of this previously undescribed leaf spot disease. The conidial morphology showed remarkable consistency with previous descriptions of *N. oryzae* [25], while molecular data from the ITS1, TUB2, and TEF1-α genes provided definitive species confirmation. Our findings highlight an emerging phytosanitary challenge for solanaceous crop production systems. The demonstrated pathogenicity of *N. oryzae* in tomato necessitates the urgent implementation of integrated disease management strategies to mitigate economic losses in affected regions. This discovery underscores the importance of continued pathogen surveillance and host range studies to address evolving threats in agricultural ecosystems.

The genus *Nigrospora* is a widely-distributed fungus that can exist as an endophyte [26]. Among some *Nigrospora* species reported in a previous study, *N. oryzae, N. osmanthi*, and *N. sphaerica* were recorded frequently as pathogenic on a broader range of host plants. The hosts of *N. oryzae* include herbaceous plants such as Asiatic dayflower (*Commelina communis*) [27] to woody plants such as Cotton rose (*Hibiscus mutabilis*) [28], and from monocotyledonous plants such as Rice (*Oryza sativa*) [11] to dicotyledonous plants such as Peanut (*Arachis hypogaea*) [29]. Even though the pathogenic behavior of *N. oryzae* is prominent, there has been no report on its infection of tomatoes to date. This represents the first confirmed report of *N. oryzae* as a pathogenic agent in tomato crops, significantly expanding its known host range within the Solanaceae family.

The spore dispersal of *Nigrospora* is aided by the wind, rain splash, and insect vectors supporting the rapid spread of the disease. The presence of a sticky mucilaginous substance was observed on discharged spores [30]. It has been hypothesized that this mucilaginous substance facilitates adherence to the host substrate or to a vector, such as mites, as a successful spore dispersal mechanism [26]. In this study, we hypothesized that microbial biofouling on cover slips might cause the conidia of *N. oryzae* to crack. We found that the conidia of *N. oryzae* cracked irregularly on plastic cover slips. This aberrant response stands in sharp contrast to the typical appressorium development observed in related species such as *Magnaporthe oryzae*. In these species, hydrophobic surfaces generally trigger the generation of turgor pressure and the formation of penetration pegs via melanized appressoria. Therefore, the absence of functional appressoria in *N. oryzae-YN* strongly implies potential defects either in the perception of environmental signals or in the downstream regulatory pathways that govern the differentiation of infection structures. Moreover, the release of lipid droplets may suggest a compromised membrane integrity or altered lipid metabolism during the failed morphogenetic processes.

To facilitate host colonization, fungal pathogens deploy an extensive array of extracellular enzymes that enable tissue invasion, immune evasion, and host ROS scavenging. Among these enzymatic arsenals, extracellular laccase and peroxidase emerge as particularly crucial virulence factors [17,31]. In this study, we found that *N. oryzae-YN* lost the extracellular laccase and peroxidase activity. Species of *Nigrospora* commonly occur as plant pathogens, endophytes, or saprobes, and have also been commonly recorded as plant pathogens on many important economic crops, fruits, and ornamentals [32]. In addition, it is noteworthy that the top three most ubiquitous *Nigrospora* species (i.e., *N. sphaerica*, *N. oryzae*, and *N. chinensis*) all belong to early divergent species in the genus [25]. We speculate that during the long evolutionary process, after *Nigrospora* has transformed from a plant endophyte into a pathogenic fungus, it can be recognized by plants in an affinity manner, allowing it to overcome the host immunity and complete the infection without the need to secrete the aforementioned extracellular proteins.

Genetic transformation stands as an indispensable tool in modern research, facilitating in-depth gene function studies and the genetic enhancement of organisms. For plant fungal pathogens like *N. oryzae*, understanding their disease-causing mechanisms is crucial, especially considering the potential threats they pose to tomato production. One of the most effective approaches to decipher the pathogenic mechanisms of these fungi is through gene disruption, either in a targeted or random fashion, to isolate mutants with altered virulence. In filamentous fungi, DNA introduction via transformation usually leads to either the heterologous (illegitimate) or homologous integration of the transforming DNA into the target genome. Homologous integration is particularly valuable as it enables targeted gene disruption by replacing the wild-type allele on the genome with a mutant allele carried by the transforming DNA. This process has been extensively utilized to elucidate the role of newly identified fungal genes in pathogenicity across numerous fungal species. While genetic transformation protocols have been well-established for many fungal species, and despite the description of the first-draft genome assembly of *N. oryzae* [6], there were no available genetic transformation methods for *N. oryzae*. This lack of methodology has hindered in-depth research on its pathogenic mechanisms. In this study, we established a PEG-mediated protoplast transformation system for *N. oryzae*, which will facilitate future investigations into the genomic features of *N. oryzae* and the pathogenic mechanism of this fungus.

Biological control represents a highly accessible, environmentally benign, and cost-effective alternative for the prevention and suppression of plant diseases. *Bacillus* species with their antimicrobial and plant-growth-promoting effects are frequently used as biocontrol agents to enhance the resilience of agricultural production against biotic stresses [24]. There have been numerous studies on the antifungal metabolites synthesized by *Bacillus subtilis* and their potential applications in disease suppression. For example, the antagonistic *Bacillus subtilis* strain NCD-2, isolated from the cotton rhizosphere, showed strong in vitro inhibition against filamentous fungi, and fengycin-type lipopeptides are the main antifungal active compounds produced by *Bacillus subtilis* NCD-2 [13]. Additionally, the organic volatile compounds produced by *Bacillus subtilis* CFFSUR-B31 have an inhibitory effect on the mycelium growth and spore germination of phytopathogenic fungi [15]. In this study, our laboratory preserved three biocontrol strains with antagonistic effects. Among them, *Bacillus velezensis* B31 exhibited the most significant antagonistic effect and could be further investigated as an effective biocontrol resource.

## 5. Conclusions

In the present study, *N. oryzae*, a pathogenic fungus renowned for its extensive host range, was successfully identified as the causative agent of tomato leaf spot disease. This represents the first worldwide report of tomato leaf disease caused by *N. oryzae*. The findings have broadened our comprehension of the host range susceptible to *N. oryzae* infection. To control the spread of *N. oryzae* and reduce the damage it causes to tomatoes, we, respectively, evaluated the antagonistic effects of *Bacillus subtilis* NCD-2, HMB19198, and *Bacillus velezensis* B31 against *N. oryzae*. The results showed that these three strains could inhibit the growth of *N. oryzae*. Furthermore, *Bacillus velezensis* B31 demonstrated great potential as a biocontrol agent (BCA) for managing tomato diseases and other plant diseases incited by *N. oryzae*. It is important that we have established a PEG-mediated protoplast transformation system of *N. oryzae*, which will be helpful for people to conduct in-depth research in the future on the pathogenic mechanism of *N. oryzae* and its interaction mechanism with hosts.

## Figures and Tables

**Table 1:** Primers used in this study.

Primer Name	Sequence (5′-3′)	Remark
TUB2-F	GGTAACCAAATCGGTGCTGCTTTC	Amplification of TUB2 sequence
TUB2-R	ACCCTCAGTGTAGTGACCCTTGGC	Amplification of TUB2 sequence
5EF1-728F	CATCGAGAAGTTCGAGAAGG	Amplification of TEF1-α sequence
EF1-986R	TACTTGAAGGAACCCTTACC	Amplification of TEF1-α sequence
ITS1	CTTGGTCATTTAGAGGAAGTAA	Amplification of ITS1 sequence
ITS4	TCCTCCGCTTATTGATATGC	Amplification of ITS1 sequence

**Table 2:** Information on isolates/strains used to construct phylogenetic trees based on the ITS sequences.

Organism	Accession	Host
*Apiosporaceae* sp.	KU747955	*Lomariopsis mexicana*
*Buergenerula spartinae*	MN644634	*Sporobolus alterniflorus*
*Chromocleista* sp.	MN644566	*Sporobolus pumilus*
*Fungal endophyte*	KY765249	*Taxodium distichum*
*Fungal* sp.	PQ320869	*Tachigali versicolor*
*Kohlmeyeriopsis medullaris*	MN644565	*Sporobolus pumilus*
*Nigrospora oryzae*	-	*Solanum lycopersicum*
*Nigrospora oryzae*	GQ221861	*Zea mays*
*Nigrospora oryzae*	GQ328855	*Zea mays*
*Nigrospora oryzae*	KX958066	*sediment below ocean floor*
*Nigrospora* sp.	MN153965	*Zea mays*
*Nigrospora* sp.	MF942937	*Dicranum scoparium*
*Nigrospora sphaerica*	KT462720	*surface sterilized cow pea seed*
*Sordariomycetes* sp.	KP991424	*Quercus hypoleucoides*
*Sordariomycetes* sp.	JQ759919	*Pinus elliottii*
*Trichoderma afroharzianum*	MN644564	*Sporobolus pumilus*

**Table 3:** The nucleotide homology analysis of ITS1/TUB2/TEF1-a.

Gene	Organism	Accession	Identity	Host
ITS1	*N. oryzae* *2684*	EU272488.1	99.43%	*Espeletia* sp.
TUB2	*N. oryzae* *GUCC:19-5152*	OP450803.1	89.60%	*Broadbean*
TEF1-a	*N. oryzae* *LC6969*	KY019386.1	72.46%	*Citrus reticulata*

## Data Availability

Data are contained within the article.
